# The Translational Geroscience Network: Supporting a New Paradigm to Alleviate Age-Related Chronic Disease

**DOI:** 10.1093/geroni/igaa057.3042

**Published:** 2020-12-16

**Authors:** Jamie Justice, Stephen Kritchevsky, George Kuchel, James Kirkland

**Affiliations:** 1 Wake Forest School of Medicine, Winston-Salem, North Carolina, United States; 2 University of Connecticut, Farmington, Connecticut, United States; 3 Mayo Clinic, Rochester, Minnesota, United States

## Abstract

Aging is the leading risk factor for many chronic diseases. Through traditional approaches to drug development and treatment focus on treating one disease at a time, the geroscience hypothesis posits that by targeting fundamental aging processes one could alleviate multiple age-related diseases. Now several geroscience-guided interventions are at the point of entering human clinical trials. To accelerate testing of this important hypothesis, an interdisciplinary Translational Geroscience Network (TGN; R33 AG061456) has recently been established. The TGN is a new national resource of aging research centers committed to working together toward complementary, small-scale, proof-of-concept “use case” clinical studies. One such pilot will be highlighted: a translational trial of senolytics, or drugs targeting the biological aging process cellular senescence in patients with idiopathic pulmonary fibrosis. The promise of geroscience provides another reason “why age matters”: by studying the basic biology of aging, we may open novel therapeutic opportunities for challenging age-related diseases.

